# Selective trace elements significantly enhanced methane production in coal bed methane systems by stimulating microbial activity

**DOI:** 10.1128/spectrum.03508-23

**Published:** 2024-01-18

**Authors:** Kuk-Jeong Chin, Burcu Ünal, Michael Sanderson, Feranmi Aboderin, Klaus Nüsslein

**Affiliations:** 1 Department of Biology, Georgia State University, Atlanta, Georgia, USA; 2 Department of Microbiology, University of Massachusetts, Amherst, Massachusetts, USA; 3 Department of Environmental Engineering, RheinMain University of Applied Sciences, Wiesbaden, Germany; University of Mississippi, University, Mississippi, USA

**Keywords:** coalbed methane, active microbial community, trace elements, *mcrA *transcript, 16S rRNA cDNA, microcosms

## Abstract

Trace elements are associated with the microbial degradation of organic matter and methanogenesis, as enzymes in metabolic pathways often employ trace elements as essential cofactors. However, only a few studies investigated the effects of trace elements on the metabolic activity of microbial communities associated with biogenic coalbed methane production. We aimed to determine the effects of strategically selected trace elements on structure and function of active bacterial and methanogenic communities to stimulate methane production in subsurface coalbeds. Microcosms were established with produced water and coal from coalbed methane wells located in the Powder River Basin, Wyoming, USA. In initial pilot experiments with eight different trace elements, individual amendments of Co, Cu, and Mo lead to significantly higher methane production. Transcript levels of *mcrA*, the key marker gene for methanogenesis, positively correlated with increased methane production. Phylogenetic analysis of the *mcrA* cDNA library demonstrated compositional shifts of the active methanogenic community and increase of their diversity, particularly of hydrogenotrophic methanogens. High-throughput sequencing of cDNA obtained from 16S rRNA demonstrated active and abundant bacterial groups in response to trace element amendments. Active *Acetobacterium* members increased in response to Co, Cu, and Mo additions. The findings of this study yield new insights into the importance of essential trace elements on the metabolic activity of microbial communities involved in subsurface coalbed methane and provide a better understanding of how microbial community composition is shaped by trace elements.

**IMPORTANCE:**

Microbial life in the deep subsurface of coal beds is limited by nutrient replenishment. While coal bed microbial communities are surrounded by carbon sources, we hypothesized that other nutrients such as trace elements needed as cofactors for enzymes are missing. Amendment of selected trace elements resulted in compositional shifts of the active methanogenic and bacterial communities and correlated with higher transcript levels of *mcrA*. The findings of this study yield new insights to not only identify possible limitations of microbes by replenishment of trace elements within their specific hydrological placement but also into the importance of essential trace elements for the metabolic activity of microbial communities involved in subsurface coalbed methane production and provides a better understanding of how microbial community composition is shaped by trace elements. Furthermore, this finding might help to revive already spent coal bed methane well systems with the ultimate goal to stimulate methane production.

## INTRODUCTION

Coalbed methane (CBM) is a form of natural gas extracted from coalbeds globally, where it is adsorbed onto the solid matrix of coal (1–3, 4–7). CBM is produced either via transformation of organic matter by geochemical processes (thermogenic gas) during the thermal evolution of coal at greater depths or via microbial degradation of coal organic molecules at shallower depths (biogenic gas) (~20% of global biogas resources) (1, 8–11). Knowledge of the microbial communities related to coalbeds is of great interest for the conversion of coal to methane (12). Numerous studies have characterized the microbial communities associated with subsurface coalbeds (4, 13–16) and the surrounding groundwater (17–22) through metagenomic and transcriptomic analysis of key molecular markers such as 16S rRNA or the methyl coenzyme M reductase (*mcrA*) gene, and the GeoChip functional gene array (16, 23–35). Yet, little is known about the prevailing microbial metabolism in subsurface coalbeds.

Coal biodegradation is an active but slow process; hence, stimulation of the rate-limiting microbial degradation processes along the coal-to-methane pathway has been extensively studied both *in situ* and *ex situ* (8, 36–40). Studies on subsurface coal biodegradation and approaches by commercial companies (e.g., Luca Technologies Inc., Next Fuel Inc., and Ciris Energy) have focused on understanding the limitations and potential enhancement of coal biodegradation through the addition of nutrients or microbial consortia with the goal of enhancing CBM production. Nutrient additions often resemble those of selective growth media used to enrich members of anaerobic heterotrophic microorganisms, and previously tested nutrients include various dissolved complex carbon substrates, nitrogen source, phosphate, vitamins, trace elements (TE), macro minerals (5, 8, 19, 21, 23, 29, 33, 41–43) and methanogenic substrate amendments *in situ* and in laboratory (2, 44). In addition, the effects of physicochemical parameters (2, 45, 46) and the physical connectivity of coal, specifically, surface area enhancement for gas adsorption, have been reported (2, 47–50). Other studies aimed to enhance methane production by providing complex nutrient sources (e.g., yeast extract, trypticase peptone, algal biomass, rice straw, emulsified nutrient solutions) (18, 36, 51–55) and bioaugmentation (19, 20, 56). A stimulation of CBM production in wells of the PRB resulted from the addition of microbes that were previously collected and concentrated from neighboring wells (57). This type of stimulation in active wells might be caused by TE contained in dead cells of a microbial community adapted to gas production in CBM wells (58).

The effects of nutrient additions on the metabolic activity of complex microbial communities are difficult to determine. The knowledge gained from repeated amendments of individual nutrients with determined concentrations could enable the development of specific and cost-effective methods for nutrient additions in coal seams (20). In general, specific TEs, as micronutrients, and their variable concentrations affect the structure and function of microbial communities (59–61). These TEs play vital roles in growth, activity, and physiology of microorganisms as critical cofactors, structural components of enzymes, and electron carriers in redox reactions (62, 63). The deficiency of TEs often results in reduced efficiency of the anaerobic metabolism, causing changes in the structure and function of microbial communities (60, 61, 64–67). The essential roles of TEs such as Mo, Co, Cu, Zn, Fe, Ni, W, and Se in an effective anaerobic digestion process and enhanced methane production have been investigated in microcosms or pilot scale reactors (16, 68–78). However, few studies have investigated the relationship between coal and TE on methane production in subsurface.

Trace elements are known to be one of the limiting chemical constituents of methanogens associated with subsurface coalbeds (22, 33), yet we have limited knowledge about the effects of essential TEs on microbial CBM production. Several studies have evaluated the relationship between *in situ* TEs in the produced water and gas production rate at CBM sites to guide the development of CBM production with a focus on hydrogeology and economics (79–82). CBM wells with low gas production are characterized by higher water removal and lower *in situ* TE concentrations compared to wells with high gas production. In the study by Guo et al. (80), lithium, gallium, rubidium, strontium, and barium were analyzed for their correlation between methane production and TE concentrations. The relationship between the concentration of TEs and the depth of the coal seam was observed in the Tiefa Basin in northeastern China, where the productivity of CBM wells decreased with the increasing Li, Sr, and Ba concentration. Changes in the distribution and concentration of TEs are affected by the depositional environmental factors, especially redox conditions, water-rock interactions, total dissolved solids (mainly sodium salts), and pH with increasing depth (81–83). A recent study described the effect of exogenous Fe^2+^ addition on the conversion of coal to methane, with the highest increase in methane production at 13.5%. The synthesis and activity of hydrogenase, which plays a role in anaerobic biodegradation pathways of coal to methane, was significantly enhanced by Fe^2+^ supplementation, but the effects of other TEs were not reported in this study (22). In a study by Wang et al. (77) in southern Junggar Basin, China, *Methanothrix* was associated with a high Mo concentration, and *Methanolobus* was closely associated with high concentrations of Li, Sc, Cs, and Mn. However, this study did not investigate the effects of essential TEs on coal-associated microorganisms or the effects of TEs on biogenic methane production.

The depositional environment and post-depositional processes are factors that affect the chemical content of subsurface coalbed water. Hence, the chemical characteristics of subsurface water vary depending on geography, hydrogeology, methane generation pathway, location, depth, and type of coal (77, 84–86). The concentrations of TEs in produced water are dependent on leaching of TEs from coal to produced water, runoff and circulation of ground water, adsorption of TEs onto coal, redox potential of the subsurface environment as well as pH, and the depth of coal formations (79, 82, 83). Because of these factors, TE concentrations are not constant either between basins, between wells, at different depths,or at different time intervals.

The aim of this study was to investigate the effects of three essential TEs, Co, Cu, and Mo, on the structure and function of bacterial and methanogenic archaeal communities, which can enhance methane production in subsurface coalbeds. TEs were individually added to microcosms containing produced water and coal from newly drilled CBM wells located in the PRB, Wyoming, USA. We hypothesized that (i) methane production will be enhanced in TE-amended microcosms, (ii) transcript levels of *mcrA* will correlate with increased methane production in TE-amended microcosms (*mcrA* encodes the α-subunit of methyl-coenzyme M reductase that catalyzes the final step in methanogenesis), and (iii) that the addition of an individual TE will affect the structure of the active methanogenic and bacterial communities.

## MATERIALS AND METHODS

### Sampling of CBM well produced water and coal

Produced water samples were collected from CBM wells located within the Tongue River member of the Fort Union Formation in the PRB near Gillette, Wyoming (Fig. S1). All produced water samples were collected from actively pumping surface pump facilities associated with the respective target wells. Not all wells were producing economically valuable quantities of gas at the time of sampling and microcosms were established with produced water from a well that did no longer produce gas in economic quantities. Produced water samples were collected after extensive pumping to clean out the well bore and obtain a formation-derived sample. A presterilized water hose was insterted into the pumpwater stream at the well head pump outflow, and the opposite end was placed into sterile, gas-tight, one-liter HDPE bottles (Thermo Fisher Scientific, Waltham, MA; previously flushed with N_2_-gas). To sample under anoxic conditions, at least three bottle volumes were replaced by overflow to ensure oxygen-free sampling. To eliminate headspace, the bottles were filled with produced water without headspace and shipped overnight to the lab on ice. On the day of arrival at the laboratory, the produced water samples were immediately processed to establish the microcosms and sample bottles were stored at 4°C in the dark until further use. A block of subbituminous coal (~3,000 cm^3^) was collected under oxic conditions from the water sampling collection area (Wyodak-Anderson coal zone) and shipped to the laboratory overnight in a gastight container at ambient temperature. The coal block was stored under anoxic conditions in the dark at 4°C until further processing (subsampling under an oxygen-free atmosphere, see section “Microcosm design”).

### Trace element selection and analysis

For an initial selection of specific TEs and identification of optimal concentrations, produced water collected from eight different CBM wells in 2008 was used to set up the test microcosms in triplicates. Of total eight TEs tested (Zn, Mn, Fe, Ni, W, Co, Mo, Cu), the amendment of three TEs, Co, Cu, and Mo, significantly enhanced methane production in the test microcosms and were selected for further analysis (Table 1). For the subsequent set-up of microcosms, produced water was collected in 2012 from three wells within the same coal formation (Well-A, Well-B, Well-C), two wells that are currently recovering economic volumes of gas, and one well from which no longer economic volumes of gas are produced. Produced water from the non-gas-producing well (Well-B) with the highest microbial cell count (2.1 × 10^7^ at >0.2 µm and 2.67 × 10^5^ at <0.2 µm) was used to set up additional microcosms. Total cell numbers in three well production samples (Well-A, Well-B, Well-C) were estimated by direct microscopic count based on cell-sized fractions (>0.2 µm and <0.2 µm) (Fig. S2). The *in-situ* water temperature of Well-B was 19 ± 0.2°C at a pH value of 7.2. The pH values fell to pH 6.9 by the end of the incubation.

**TABLE 1:** Determination of TEs and their concentration ranges that enhanced methane production^*c*^

	Trace element concentrations (µg/L) and relative methane production															
	Mo		Co		Cu		Zn		Mn		Fe		Ni		W	
*In situ* (*n* = 3)^*a*^	14.6 (±0.8)		1.1 (±0.3)		2.9 (±0.2)		305.8 (±74)		17.3 (±1.5)		20.8 (±1.8)		79.5 (±1.3)		BDL (˂1)	
Concentrations		CH_4_^*b*^		CH_4_		CH_4_		CH_4_		CH_4_		CH_4_		CH_4_		CH_4_
[I]	14.3	(+)	45.0	(++)	0.8	(−)	30.0	(−)	25.0	(−)	58.5	(−)	5.0	(−)	2.7	(−)
[II]	143.0	(++)	450.0	(+)	7.5	(+)	300.0	(−)	250.0	(−)	5,850.0	(−)	50.0	(−)	27.0	(−)
[III]	286.0	(−)	900.0	(−)	15.0	(++)	600.0	(−)	500.0	(−)	11,700.0	(−)	100.0	(−)	54.0	(−)
[IV]	1,430.0	(−)	4,500.0	(−)	75.0	(−)	3,000.0	(−)	2,500.0	(−)	58,500.0	(−)	500.0	(−)	270.0	(−)

^*a*^ Standard deviation of *in situ* TE concentrations is shown in parenthesis.

^*b*^ (+), (++), methane production significantly higher than unamended microcosms (*P* < 0.05). (−), methane production was less than that of unamended microcosms (*P* > 0.05). (−), methane production was completely inhibited. BDL, below detection limit.

^*c*^ Eight individual trace elements (Mo, Co, Cu, Zn, Mn, Fe, Ni, and W) were added to microcosms prepared in triplicate with produced water (Well-B) and coal from the Powder River Basin, Wyoming. Each microcosm was amended with four different concentrations ([I] – [IV]) of a TE. Amendments with Fe, Mn, Ni, W, and Zn either did not lead to a significant increase in methane production (*P* < 0.05) in the tested concentration range or caused complete inhibition of methane production possibly due to toxic effects. The most effective trace elements (Co, Cu, and Mo) and their specific concentrations (*P* < 0.05; shaded cells) were determined based on their cumulative methane production in the amended microcosms and these microcosms [indicated as (++)] were used for subsequent microbial analysis.

Prior to TE analysis subsamples of fresh *in situ* produced water were sequentially filtered through decreasing pore sizes from 0.45 to 0.22 µm and acidified to pH < 2 with 1% nitric acid (trace metal grade, Fisher Scientific, Pittsburgh, PA). Trace element concentrations of 22 different elements in filtered produced water were analyzed in triplicate by inductively coupled plasma-mass spectrometer (ICP-MS, Perkin Elmer Elan 9000) at the Department of Civil and Environmental Engineering, University of Massachusetts Amherst, Amherst, USA. All glassware and caps which were used in this study were initially cleaned, acid-washed in 10% HNO_3_ for 24 h, rinsed with deionized water, and baked at 450°C for 6 h to remove all traces of organic carbon.

### Microcosm design

The microcosms were set up by adding 10 mL of produced water and 1 g of crushed coal and a single TE solution (Co, Cu, or Mo) in triplicate in anaerobic Balch tubes ( Fig. S3). The control microcosm (unamended) contained the same proportion of coal and produced water as the amended microcosm but did not contain any TE solutions. To establish the TE-amended microcosms, different TEs concentration ranges (Table 1) were selected based on the average *in situ* concentrations of TEs in 11 CBM-produced water samples (Table S1). An outer layer of approximately 5 cm of coal was removed from a large block using a surface-sterilized core drill, and the inner section was sampled for the set-up of microcosms. Coal was crushed with a sterile mortar and pestle set and then sieved to a desired size range (600–850 µm in diameter). All coal manipulations were performed in an anaerobic chamber (Vacuum Atmospheres, Hawthorne, CA) under oxygen-free conditions. Coal was the only organic carbon source added. Tubes were crimp-sealed with butyl rubber stoppers (Bellco Glass, Vineland, NJ), and the headspace atmosphere was replaced with N_2_/CO_2_ (80:20, vol/v)ol to ensure an anaerobic environment. Microcosms were incubated in the dark without agitation at *in situ* well temperature for up to 4 months.

### Methane production analysis

The headspace of the microcosms was monitored and quantified by gas chromatography using a GC-17A (Shimadzu, Co., Kyoto, Japan) equipped with an Equity-1 column (30 m × 0.53 mm ID, 3.0 µm; Supelco, Bellefonte, PA) and a flame ionization detector with helium as the carrier gas. The injector and column temperatures were set at 100°C and the detector was held at 125°C. Certified standard CH_4_ (Fisher Scientific, Pittsburgh, PA) was used for calibration. The cumulative methane concentration in the headspace of the microcosms was calculated using an empirical calibration curve. The highest methane-producing microcosms were selected for subsequent experiments. To account for the amount of methane that would be desorbed from the added coal, parallel incubations of biotic and abiotic controls were set up with 25 g of powdered coal in 25 mL of produced water under a N_2_ headspace (30 mL headspace in a 60-mL bottle). The negative control contained produced water that was filter sterilized (Fig. S4).

### Microbial biomass measurement by direct cell count

Microbial biomass in produced water was determined by direct microscopic counting. Total bacterial and archaeal cell numbers were estimated by 4′6-diamidino-2-phenylindole (DAPI) staining of nucleic acid (Sigma-Aldrich, St. Louis, MO) in three CBM-produced water samples (Well-A, Well-B, Well-C). The average number of cells in the samples was calculated by examining a sequence of 40 randomly selected microscopic fields on each slide at 1,000× oil-immersion magnification using an epifluorescence microscope (Nikon Eclipse E400, Melville, NY) equipped with a digital camera (Hamamatsu, Bridgewater, NJ). Well B was selected from three wells sampled based on the highest biomass in its produced water.

### Extraction of total RNA and mRNA

Total RNA and mRNA were extracted from two samples obtained from unamended, Co-, Cu-, and Mo-amended microcosms, respectively, after 4 months of incubation, using the protocols described by Ünal et al. (33). The isolated RNA samples were pooled together and used for cDNA synthesis.

### Reverse transcription PCR and real-time quantification of 16S rRNA and mRNA transcripts

cDNA synthesis was performed with the *mcrA*-specific reverse primer *mcrA*-rev (87) and 16S rRNA gene reverse primer 806R (88), 0.5 mg template mRNA or total RNA, and Multi Scribe MuLV reverse transcriptase (200 U; Life Technologies, Foster City, CA, USA) incubated at 25°C for 20 min followed by at 37°C for 120 min, and enzyme inactivation at 80°C for 5 s. The cDNAs generated with *mcrA*-specific or 16S rRNA gene primers were quantified with quantitative real-time PCR (qRT-PCR), using SYBR Green as described previously (33). For *mcrA*, two runs of qRT-PCR with a primer set of *mlas*/*mcrA-rev* were performed (two experimental replicates), and for each qRT-PCR run, three replicates of cDNA samples were used. For 16S rRNA, three runs of qRT-PCR with a primer set of 16S rRNA 515F/806R were performed (three experimental replicates), and for each qRT-PCR run, three replicates of cDNA samples were used.

### mcrA cDNA clone library analysis

The purified *mcrA* RT-PCR amplicons from unamended, Co-, Cu-, Mo-amended microcosm samples were ligated using the CloneJET PCR cloning kit (Thermo Fisher Scientific, Waltham, MA) according to the manufacturer’s instructions and transformed into TOP 10 chemically competent *Escherichia coli* cells (One Shot TOP10, Invitrogen). Clones were randomly selected, and plasmid inserts were verified by PCR amplification with M13F/M13R primers. Approximately 35 positive plasmids from each clone library were sequenced with the M13F primer by Sanger sequencing. Since the *mcrA* clone library was not analyzed by high-throughput sequencing but by Sanger sequencing of individual clone inserts, our analysis was limited to a low-sequencing depth. Sequence chromatograms were manually checked and edited for ambiguous bases by using the Chromos sequence viewer software, and sequences were aligned using ClustalX (89). Four clone libraries were grouped into operational taxonomic units (OTUs), based on 89% sequence identity cutoff (87) by using MOTHUR (90). Representative sequences from each OTU were identified using the BLAST search engine against the NCBI nucleotide sequence database (http://blast.ncbi.nlm.nih.gov/Blast.cgi).

### 16S rRNA analysis via high-throughput sequencing

16S rRNA RT-PCR amplicons generated with the universal bacterial/archaeal primer pair 515 F-806R were used for 454 high-throughput sequencing performed on the 454 GS FLX Sequencer (454 Life Sciences, Bradford, CT, USA) at the Michigan State University Research Technology Support Facility. The multiplexed fastq files generated with the 454-sequencing output (fasta and qual files) (91) were imported into QIIME2. Demultiplexed sequences were then generated with the “cutadapt demux-single” command (92) which uses the adaptor barcode sequences provided to assign sequences to individual samples and by trimming off the adaptor from the sequences. Denoising and quality control were performed using the DADA2 (93) plug-in tool in QIIME2. In the next steps, chimeric sequences were first filtered out to generate the representative sequences for amplicon sequence variants, and counts of unique sequences (ASVs) for each sample were computed to generate a feature table. Two QC methods were tested, and the method retaining more sequences was selected. Trimming of the raw sequences was performed where the sequence median quality dropped below a threshold of 20 because a quality score of 20 translates to an error probability of 1 in 100, meaning that there’s a 99% chance the base is called correctly. Chimeric sequences were then filtered out to produce the representative sequences for amplicon sequence variants (ASVs, also called features) present in the sample.

Alpha rarefaction plots were calculated to observe whether the richness of the sample groups (metadata category) was captured after filtering out sequences with low-quality scores in the quality control step. In QIIME2, at a sequencing depth of 5,000, the following four alpha-diversity metrics were calculated: Shannon entropy, Observed features, Faith’s phylogenetic diversity, and Pielou’s evenness. The flattening out of the alpha rarefaction plots of various alpha diversity metrics illustrates the adequacy of the sequencing depth for representing the diversity within the samples (Fig. S5). The taxonomy of the representative sequences was assigned through the q2 feature-classifier using a pretrained NB classifier based on Silva vs 138. Ninety-nine percent of all OTUs were full-length sequences (94, 95). Taxa abundance tables at the genus- and family-level were then exported for the generation of taxonomic composition data. Sequences classified as Archaea were filtered out from the taxonomic bar plot as 515 F-806R primer pair can sometimes have amplification biases that can impact the detection of specific archaeal clades as reported by Parada et al. (96).

### Statistical analysis

Statistical comparisons were performed using one-way ANOVA followed by pairwise multiple comparison procedures (Holm-Šídák method) at a probability level of *P* < 0.05. Error bars represented three replicates of microcosms for methane production and biological replicates for cDNA-qPCR.

## RESULTS

### Co, Cu, and Mo amendments enhanced methane production in an anaerobic coal biodegradation environment

**Fig 1:**
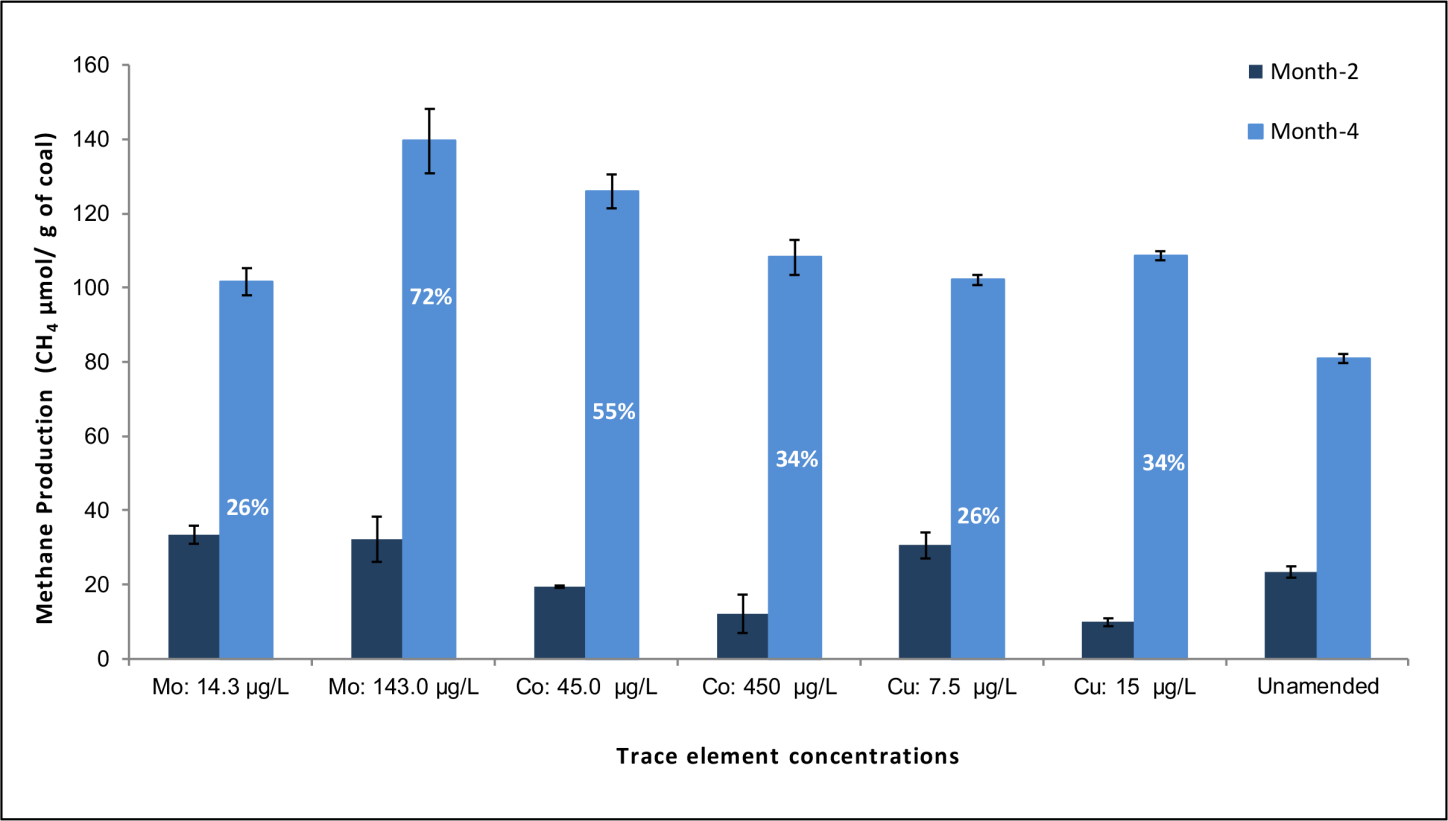
Enhanced methane production over time in coalbed microcosms with Mo, Co, and Cu amendments. Cumulative methane production was significantly increased over time in subsurface coalbed microcosms amended with varying concentrations of Co, Cu, or Mo (shown for time points after 2 and 4 months). Values above the Month-4 bars indicate the percent increase in methane production in response to trace element amendments compared to unamended microcosms. Methane production in all microcosms at time zero was below the detection limit. Error bars represent one standard deviation of measurements from triplicate of each set of microcosms. Mo and Co (*P <* 0.001) and Cu (*P <* 0.05).

TE amendment resulted in enhanced cumulative methane production in microcosms at the well *in situ* temperature (20°C) after 2 and 4 months of incubation (Fig. 1). The addition of each of the three specific TEs (Co, Cu, and Mo) resulted in significant methane production at two selected concentrations (*P* < 0.05) (Table 1). Cumulated methane production was significantly increased in microcosms amended with Co (45.0 µg/L) (*P* < 0.001) by 55%, Cu (15.0 µg/L) by 34% (*P* < 0.05), and Mo (143.0 µg/L) by 72% compared to unamended microcosms (6.8 µg/L) (*P* < 0.001) (Fig. 1). The TE amendments are 45 times higher than the *in situ* concentration of Co (1.1053 µg/L ± 0.3), 5 times higher than the *in situ* concentration of Cu (2.9 µg/L ± 0.2), and 10 times higher than the *in situ* concentration of Mo (14.6 µg/L ± 0.8) (Table 1). Methane production in all microcosms at time zero was below the detection limit. No methane production was detected during the incubation period in any of the negative control microcosms. The addition of the TEs Fe, Mn, Ni, W, and Zn at above or below their *in-situ* concentrations either did not cause a significant enhancement in methane production (*P* > 0.05) or caused complete inhibition of methane production (Table 1).

### Active microbial community shifted after Co, Cu, and Mo amendment

**Fig 2:**
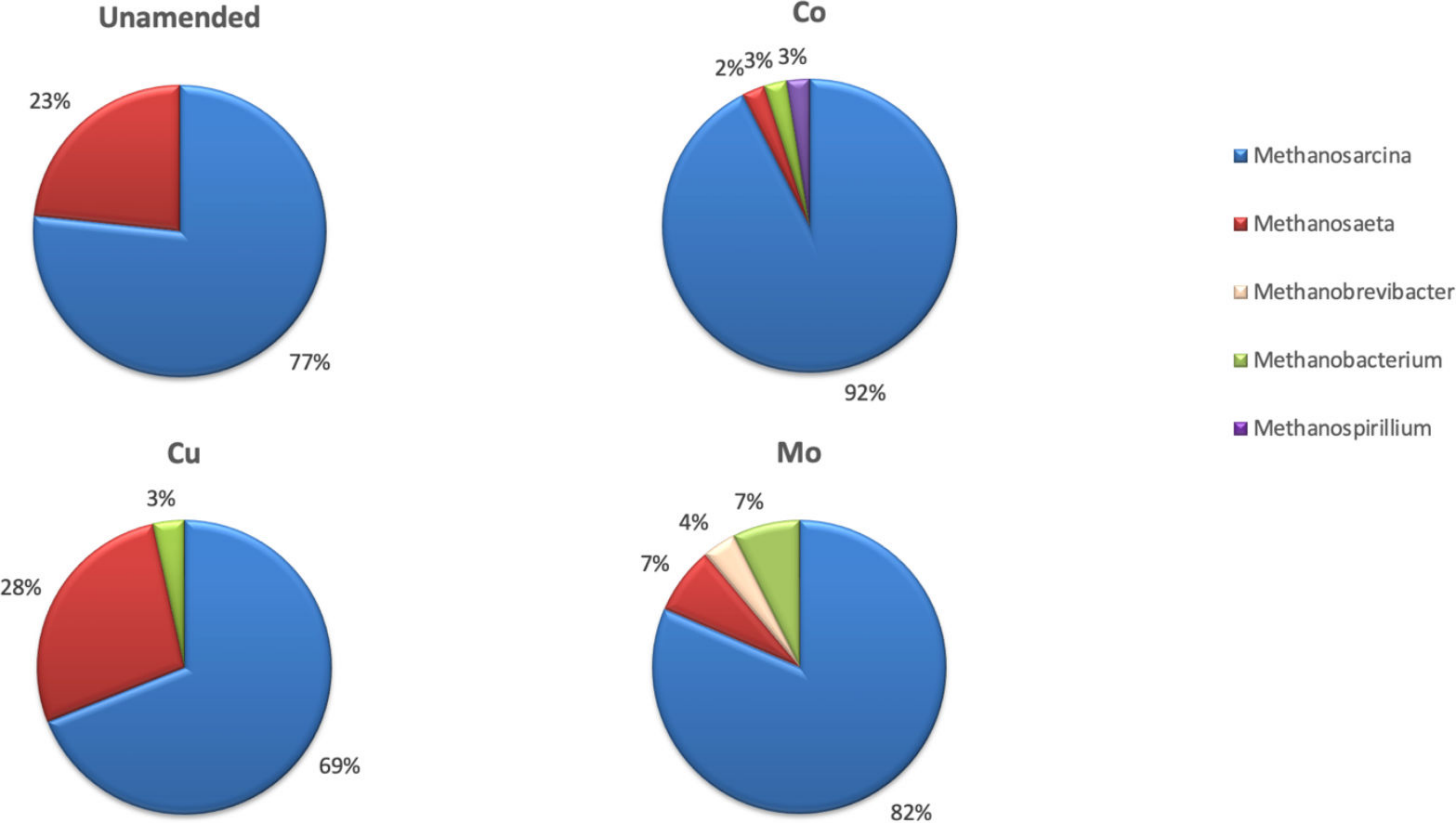
Shift of the metabolically active methanogenic community composition in response to trace element amendment in CBM microcosms. After 4 months of incubation, the phylogenetic composition of metabolically active methanogens was analyzed at genus level in Co, Cu, and Mo amended microcosms and unamended control microcosms. The pie chart illustrates the relative contribution of diverse methanogenic members belonging to different genera based on the *mcrA* cDNA clone sequence similarity.

The effects of TEs on the microbial community composition and on the expression levels of both *mcrA* and bacterial 16S rRNA genes were analyzed from microcosms that exhibited the highest methane production [Co (45 µg/L), Cu (15 µg/L), and Mo (143.0 µg/L)] (Fig. 1). Four cDNA clone libraries of *mcrA* transcripts were constructed with *mcrA* RT-PCR amplicons from Co-, Cu-, and Mo-amended and unamended microcosms, respectively. Approximately 35 plasmids from clones randomly selected from each clone library were sequenced. The results of the cDNA clone library analysis demonstrated a higher diversity of the methanogenic community in TE-amended microcosms compared to unamended microcosms (Fig. 2). Methanogenic members closely related to the genera *Methanosarcina* (77%) and *Methanosaeta* (23%) were metabolically active and dominant in unamended microcosms. Members of acetoclastic *Methanosaeta* and *Methanosarcina* utilizing acetate, H_2_, and methylated compounds were also active in all TE-amended microcosms but at varying abundances (Fig. 2). The amendment of Co, Cu, and Mo increased the diversity of the methanogenic community compared to the unamended microcosms, specifically hydrogenotrophic methanogens (*Methanobacterium, Methanobrevibacter, Methanosprillum*), and resulted in a shift of the metabolically active community composition (Fig. 2). In Co-amended microcosms, members of the genus *Methanosarcina* increased (92%) while the members of the genus *Methanosaeta* significantly decreased compared to ones in the unamended microcosms. Active members of the hydrogenotrophic genera *Methanobacterium* (3%) and *Methanospirillum* (3%) were also detected. In the Cu-amended microcosms, members of the genus *Methanosarcina* decreased (69%) and members of the genus *Methanosaeta* increased (28%) compared to the unamended microcosms. Members of the genus *Methanobacterium* (3%) were also metabolically active. In the Mo-amended microcosms, the members of the genus *Methanosarcina* increased (82%), while the members of the genus *Methanosaeta* significantly decreased compared to those in the unamended microcosms. In addition, active members of the hydrogenotrophic genera *Methanobacterium* (7%) and *Methanobrevibacter* (3%) were detected (Fig. 2).

**Fig 3:**
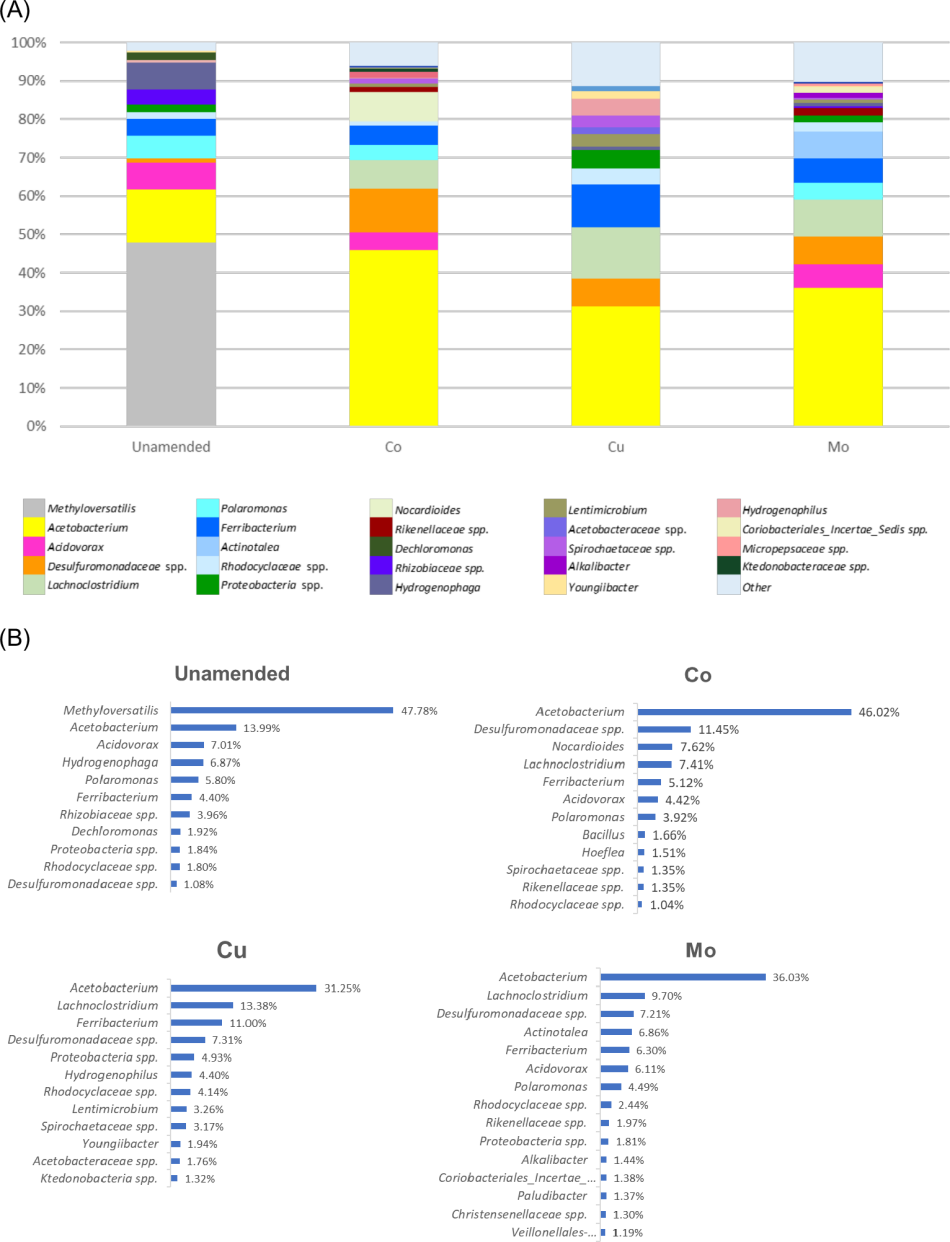
Relative abundance of bacterial groups in unamended and trace element- amended CBM microcosms. (**A**) Bacterial composition by relative abundance; (**B**) bacterial group greater than 1% of the active community.

Analyses of 16S rRNA cDNA sequences demonstrated a significant shift in the structure of the active bacterial communities in the TE-amended microcosms compared to the unamended microcosms (Fig. 3). The unamended microcosms were dominated by members of the genus *Methyloversatilis* (47.8%). Interestingly, members of the genus *Methyloversatilis* were not detected in any of the TE-amended CBM microcosms. The amendment of TEs resulted in a notable increase of the members of the genus *Acetobacterium* which were dominant in all TE-amended CBM microcosms. The TE amendments also resulted in a significant increase of the members of the family *Desulfuromonadaceae* and the genus *Lachnoclostridium* compared to unamended microcosms. In Cu-amended microcosms, the members of the genera *Acetobacterium* (31.3%) and *Lachnoclostridium* (13.4%) were the most abundant and active compared to unamended microcosm communities. Co-amendment resulted in an increase of members of the families *Acetobacterium* (46%) and *Desulfuromonadaceae* (11.5%) compared to unamended microcosms. Members of the genera *Acetobacterium* (36.0%) and *Lachnoclostridium* (9.7%) were the most abundant and active in the Mo-amended microcosms.

Analyses of the 16S rRNA cDNA sequences also revealed that Cu-amended microcosms differed in active and abundant microbial groups compared to Co- and Mo-amended microcosms (Fig. 3). Members of the genera *Acidovorax* and *Polaromonas* were abundant in unamended, and Co- and Mo-amended microcosms but were not detected in microcosms supplemented with Cu, while members of the genus *Actinotalea* became active and abundant only in Mo-amended microcosms. The values of Shannon entropy, Faith’s phylogenetic diversity (PD), and observed features were significantly higher in Cu-amended microcosms compared to unamended, Co- and Mo-amended microcosms (Fig. 6A through C). Pielou evenness values were relatively similar in all microcosms with and without amendment of TEs (Fig. 6D).

### Trace element amendment enhanced methanogenic activity

**Fig 4:**
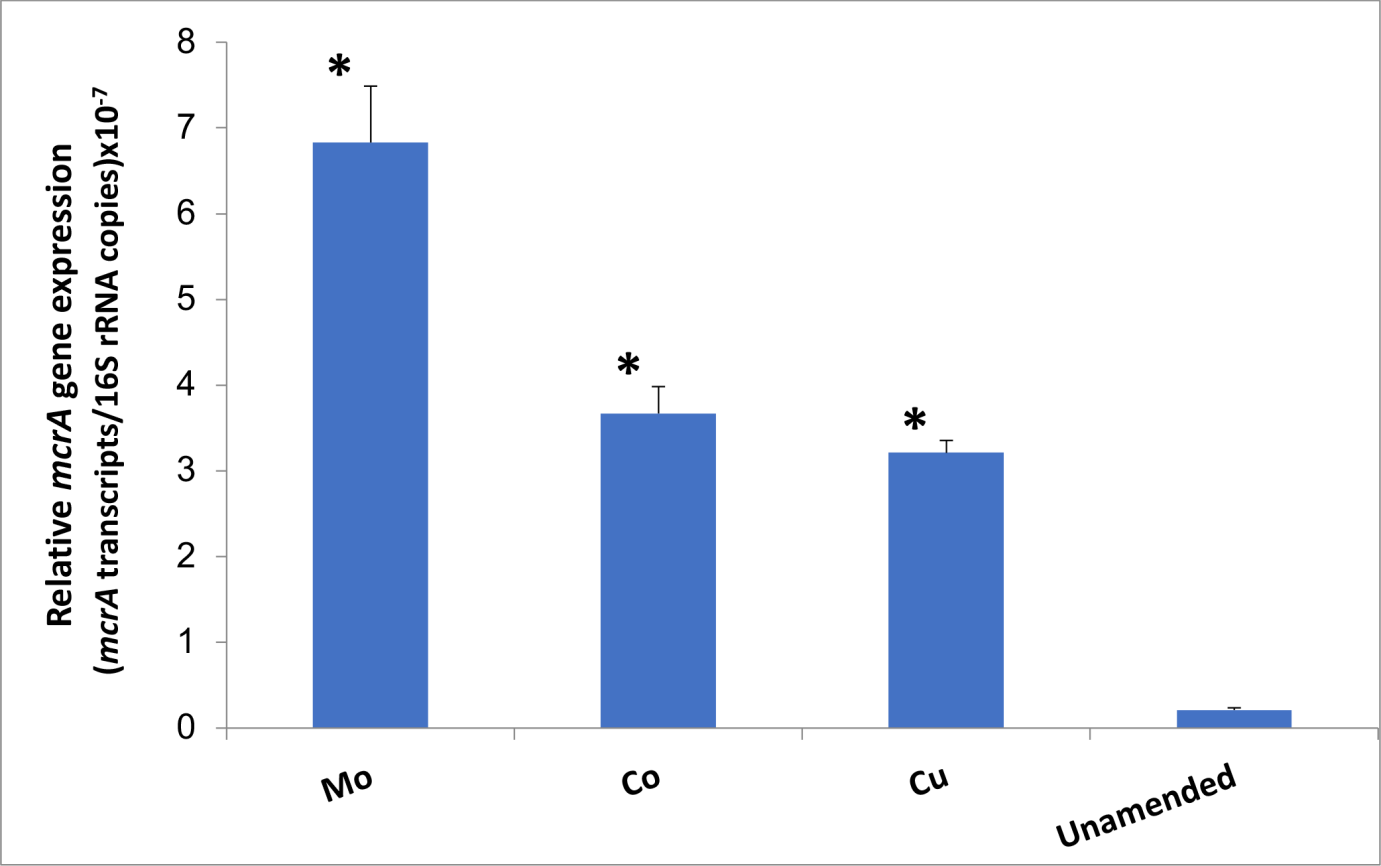
Relative expression of *mcrA* increased approximately 15- to 33-fold in CBM microcosms amended with trace elements (Co, Cu, and Mo) compared to unamended microcosms. Copies of *mcrA* transcripts were normalized to copies of total 16S rRNA transcripts. Multiple pairwise comparisons were tested using the statistical method by Holm-Šídák. The asterisk (*) above the error bars indicates a significant difference (*P* < 0.05) between means of the TE-amended and unamended control microcosms. Data represent means ± SD of the ratio of triplicate qRT-PCR determinations on a pooled sample from duplicate microcosms.

To understand the effect of each TE on the activity of the methanogenic community, the levels of *mcrA* transcripts were quantified as proxies for methanogenesis (25). The amendment of TEs resulted in a significant increase of *mcrA* transcript levels compared to unamended microcosms (*P* < 0.05) (Fig. 4). The relative expression of *mcrA* (the ratio of *mcrA* transcripts to 16S rRNA copies) significantly increased in TE-amended coalbed microcosms (Co, Cu, and Mo) compared to unamended microcosms (Fig. 4). Amendments of Co, Cu, and Mo resulted in 17.5, 15.3, and 32.5 times higher relative expression of *mcrA* compared to unamended microcosms, respectively. The level of *mcrA* in Mo-amended microcosms was higher compared to ones in Co- and Cu-amended microcosms (*P* < 0.05) (Fig. 4).

**Fig 5:**
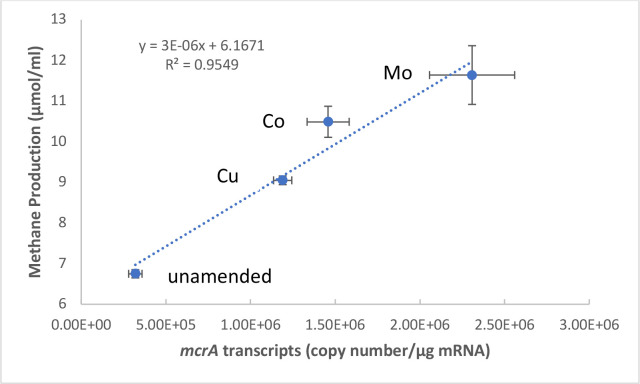
Cumulative methane production correlated (*R*^2^ = 0.95) with the transcript levels of *mcrA* in Co-, Cu-, and Mo-amended CBM microcosms after 4 months of incubation compared to unamended microcosms. Error bars show standard deviations from experimental series.

These results correspond well to significantly increased levels of methane production in TE-amended microcosms (Mo > Co > Cu). The methane production was positively correlated with the transcript level of the *mcrA* (Fig. 5). This demonstrates that the addition of TEs enhances the metabolic activity of the methanogenic microbiome in CBM-produced water, which might be stimulated by the increased availability of essential TEs.

## DISCUSSION

This study investigated the effects of three biologically essential TEs (Co, Cu, and Mo) on microorganisms associated with subsurface coalbed methane production in the PRB, Wyoming, USA. To understand the responses of microbial communities in CBM systems to three individual TE supplements, methane production and the levels of *mcrA* transcripts were determined. Furthermore, the structures of active bacterial and methanogenic communities in unamended microcosms were compared to ones in TE-treated microcosms.

Cumulative methane production in all three TE-treated microcosms was significantly higher than in parallel microcosms that remained unamended. These findings suggest that the microbial community capable of producing methane was either deficient in Co, Cu, and Mo or required higher concentrations of these TEs. Deficiency, or suboptimal availability of TEs impacts cell physiology and results in an altered, often reduced metabolic activity (97). Commonly known essential TEs such as Fe, Ni, or Zn were present in sufficient amounts in the CBM system investigated in this study.

In the present study, microcosms were set up by adding crushed coal to compensate for the low concentrations of bioavailable nutrients in the produced water. Crushed coal increases the surface area of coal in microcosms enabling an enhanced interaction among coal, microorganisms, and TEs. However, the microbial community in microcosms still lacked sufficient concentration of TEs for optimal methane production which suggested the supplementation of specific TEs (Co, Cu, and Mo) is essential.

### Effects of TEs on biogenic methane production associated with coalbed basins

Typically, the concentrations of TEs in the composition of the coal are significantly higher than in the subsurface water associated with the same coalbed (98). Ulrich and DeBruyn (99) evaluated TE loss in CBM-produced water under abiotic conditions by comparing coal slurries incubated with and without coal. In the presence of coal, the major TEs lost from the aqueous phase were determined to be Co, Cu, and Mo. This result is in good agreement with our results, which showed that the cumulative methane production in all three TE-amended microcosms (Co, Cu, and Mo) was significantly higher than in unamended microcosms (Fig. 1). These results suggest that the methane-producing microbial community is deficient in Co, Cu, and Mo and requires adequate concentrations of these TEs.

Trace element deficiency affects cell physiology and results in altered, often reduced, metabolic activity (97). Supplementation of Fe, Mn, Ni, W, and Zn (commonly known essential TEs) in our microcosms either did not enhance methane production (*P* > 0.05) in the concentration ranges tested or caused complete inhibition of methane production, possibly due to toxic effects (Table 1). Glass and Orphan (100) reported that methanogenesis in pure and mixed cultures, and even in environmental samples, is limited by the presence of insufficient amounts of bioavailable Fe, Ni, and Co. Although Fe and Ni are essential TEs for methanogenesis, the amount of Fe and Ni [20.8 (±1.8) and 79.5 (±1.3) µg/L, respectively] in the produced water of the sampled coalbed methane well (Well-B) (Table 1) may be sufficient for the metabolic activities of the current microbial communities. In fact, it was observed that the elevated concentrations of these metals inhibited methane production in the microcosms.

While an excess of available TEs could be toxic, insufficient TEs could be limiting factors for microbial function (Chen *et al*., 2019, 101). Our results demonstrated that amendments of Co, Cu, and Mo significantly enhanced methane production suggesting the strategic addition of *in situ* TE amendments as a promising approach to stimulate CBM wells to higher productivity. On the contrary, supplementation of Fe, Mn, Ni, W, and Zn in microcosms either did not enhance methane production (*P* > 0.05) in the tested concentration ranges or caused complete inhibition of methane production, possibly due to toxic effects (Table 1). Any TE requirements or potential toxic dosage effects might be specific to the geochemical origin of the particular CBM well types we had tested. The depositional environment and post-depositional processes are factors that affect the chemical content of subsurface coalbed-produced water. Therefore, the chemical characteristics of subsurface water vary with geography, hydrogeology, methane formation pathway, location, depth, and coal type (77, 84–86). The concentrations of TEs in produced water depend the on leaching of TEs from coal to produced water, groundwater recharge and circulation, the adsorption of TEs to coal, the redox potential of the subsurface environment, and the pH and the depth of coal formations (80, 82, 83 ). Due to these factors, TE concentrations are not constant between basins, or between wells, or at different depths, or at different time intervals. Considering these factors, *in situ* application approaches can be planned.

### Co, Cu, Mo as essential trace elements for methanogenic activity in CBM environments

A considerable number of studies have analyzed the microbial communities associated with terrestrial subsurface coalbeds and their limiting factors (e.g., low concentrations of electron donors, and macro- and micronutrients) (9, 102–104), and these findings have contributed to our understanding of the metabolic limits of life and energy turnover in coalbed ecosystems. Previous isotopic studies of coalbed-produced gases (δ^13^C values of CH_4_ and CO_2_) reported that hydrogenotrophic methanogenesis is the dominant pathway of methane formation in the PRB (1, 105). In contrast, several CBM-produced water enrichment studies of samples from PRB reported a predominance of acetoclastic methanogenesis (2, 10).

In this study, we determined the metabolically active methanogenic communities which are involved in hydrogenotrophic and acetoclastic methanogenesis in microcosms with different TE additions. Quantification of *mcrA* transcript levels indicated how each of the three TEs (Co, Cu, Mo) affected methanogenic activities. These results suggest that TE deficiencies in coalbed-associated methanogenic communities may limit methane production. The positive correlation of methane production (μmol/mL) with *mcrA* transcript levels (copy number/μg mRNA) was significant (*R*^2^ = 0.95) in all Co-, Cu-, and Mo-amended microcosms. The relative expression of *mcrA* increased approximately 15–33 times in TE-amended coalbed microcosms (Co, Cu, and Mo) compared to unamended microcosms (Fig. 4). The addition of Mo resulted in the highest relative expression of *mcrA*, which is approximately 33-fold higher than the unamended control and is significantly higher than those observed in Co- and Cu-amended microcosms (*P* < 0.05). These results correspond well with the significantly increased levels of methane production in TE-amended microcosms (Mo > Co > Cu) (Fig. 1). The TE-induced methane production was positively correlated with the levels of *mcrA* transcripts, indicating a direct relationship between TE bioavailability and methane production. This suggests that the *mcrA* transcript levels can be used as a proxy to predict the current methane production of metabolically active methanogenic communities associated with coalbeds in response to TE amendments.

TEs play vital roles in growth, activity, and physiology of microorganisms as critical cofactors, structural components of enzymes, and electron carriers in redox reactions (Table S2). The addition of Co resulted in the highest increase of the members of the genus *Methanosarcina* among the methanogens compared to the unamended control and the addition of Cu and Mo (Fig. 2). Cobalt is mainly found in the form of vitamin B12, a cofactor involved in methyl group transfer and rearrangement reactions in methanogenesis and homoacetogenesis (106). Several pure culture studies have reported the dependency of *Methanosarcina* spp. on Co. The metabolic activity of *Methanosarcina barkeri*, a methanol utilizing archaeon, depends mainly on the availability of Co and Mo, and methane production from an acetate-utilizing *Methanosarcina* strain was stimulated by the addition of Co (required as corrinoid Factor III) (107, 108). A methanogenic community from an anaerobic fixed-film digester was stimulated by additions of Ni, Co, and Mo additions which increased methane production by 42% (109). The higher Co requirement of methanol-utilizing methanogens and acetogens (110) is due to their unique corrinoid-containing enzymes and coenzymes (100, 111). In a mixed microcosm from an anaerobic sludge blanket reactor, methylotrophic methanogens showed a 60-fold higher affinity for methanol than acetogens. However, if both Co and methanol concentrations were high enough, acetogens could outcompete methanogens (110).

Methanol-utilizing bacteria produce either acetate or H_2_/CO_2_, which support acetoclastic or hydrogenotrophic methanogenesis. We predict that in Co-amended microcosms, acetogens outcompete methanogens and efficiently convert methanol to acetate. Increased acetate concentrations would provide a growth advantage to faster growing *Methanosarcina* over *Methanosaeta* in our microcosms. In addition, increased H_2_ and CO_2_ production from methanol degradation might stimulate the growth of hydrogenotrophic methanogens, and we found active members of *Methanobacterium* and *Methanospillium* in the Co-amended microcosms. Direct stimulatory effects of Co on methanogens were observed in a pure culture study (107). The growth of an acetate-utilizing *Methanosarcina* strain was enhanced by the presence of Co in a basal medium. Thus, the increase in the abundance of active *Methanosarcina* members observed in our study might be caused by the stimulating effect of Co on *Methanosarcina* spp.

### Microbial community shifts in response to TE amendment

Microbial community structures and associated metabolic activities are directly influenced by the physicochemical properties of the coal microenvironment. Therefore, variations in the composition, diversity, and metabolic potential of CBM-associated microbial communities differ among coal beds, well fields, and even individual wells within the same field (at different time intervals and depths) (9).

In this study, the individual amendment of essential TEs Co, Cu, and Mo significantly changed the composition and diversity of methanogenic and bacterial communities in the CBM microcosms (Fig. 2 and 3). The significant shift in microbial community structure caused by increased concentrations of each TE suggests that bioavailable TE concentrations in the environment are an essential driving factor for both shaping both microbial community structure and metabolic activity.

The phylogenetic analysis of sequences retrieved from *mcrA* cDNA clone libraries demonstrated that the members of the genus *Methanosarcina* were metabolically active and dominant methanogens in both TE amended and in unamended microcosms but in different proportions (69%–92% of the clone library) (Fig. 2). Barnhart et al. (112), in a study of enrichment cultures incubated with PRB coal, determined through archaeal 16S rRNA gene analysis that *Methanosarcina* was the most abundant group (91%), followed by *Methanospirillum* at 9%. Our results were consistent with this finding, suggesting that *Methanosarcina* (hydrogen, acetate, and methylated compound users) is also the most active and abundant methanogenic group in the PRB, Wyoming (Fig. 2). We found that their relative abundance increased (up to 28%) in Mo- and Co-amended microcosms. This increase in active *Methanosarcina* resulted in higher methane production by 72% and 55% compared to the control, respectively. Our results correspond to the findings of Glass and Orphan (100), who reported that higher concentrations of Mo and W were required for CO_2_ reduction and methylotrophic pathways. In this study, the relative abundance of metabolically active members of the *Methanosarcina* increased to 82% in Mo-amended microcosms compared to 77% in unamended control microcosms.

In this study, the members of *Methanospirillum* were observed only within Co-amended microcosms (3%) but neither in unamended nor Mo and Cu amended microcosms. *Methanospirillum* is a hydrogenotrophic methanogen and can utilize only H_2_ or formate as substrate. Growth of *Methanospirillum hungatei* on formate or hydrogen requires either tungsten (W) or Mo as an essential TE although the highest growth rates are achieved with W (113). In this study, the headspace provided for the microcosms was replaced with N_2_/CO_2_ (80:20, vol/vol) to ensure that the primary substrates for both bacteria and methanogenic communities originated from coal. In this anaerobic system, hydrogenotrophic methanogens can obtain H_2_ from syntrophic acetogens and fermenters. *Methanobacterium* (hydrogen and formate users) and *Methanobrevibacter* (hydrogen user) which were not detected in control microcosms, appeared in 7% and 4%, respectively, in Mo-amended microcosms.

**Fig 6:**
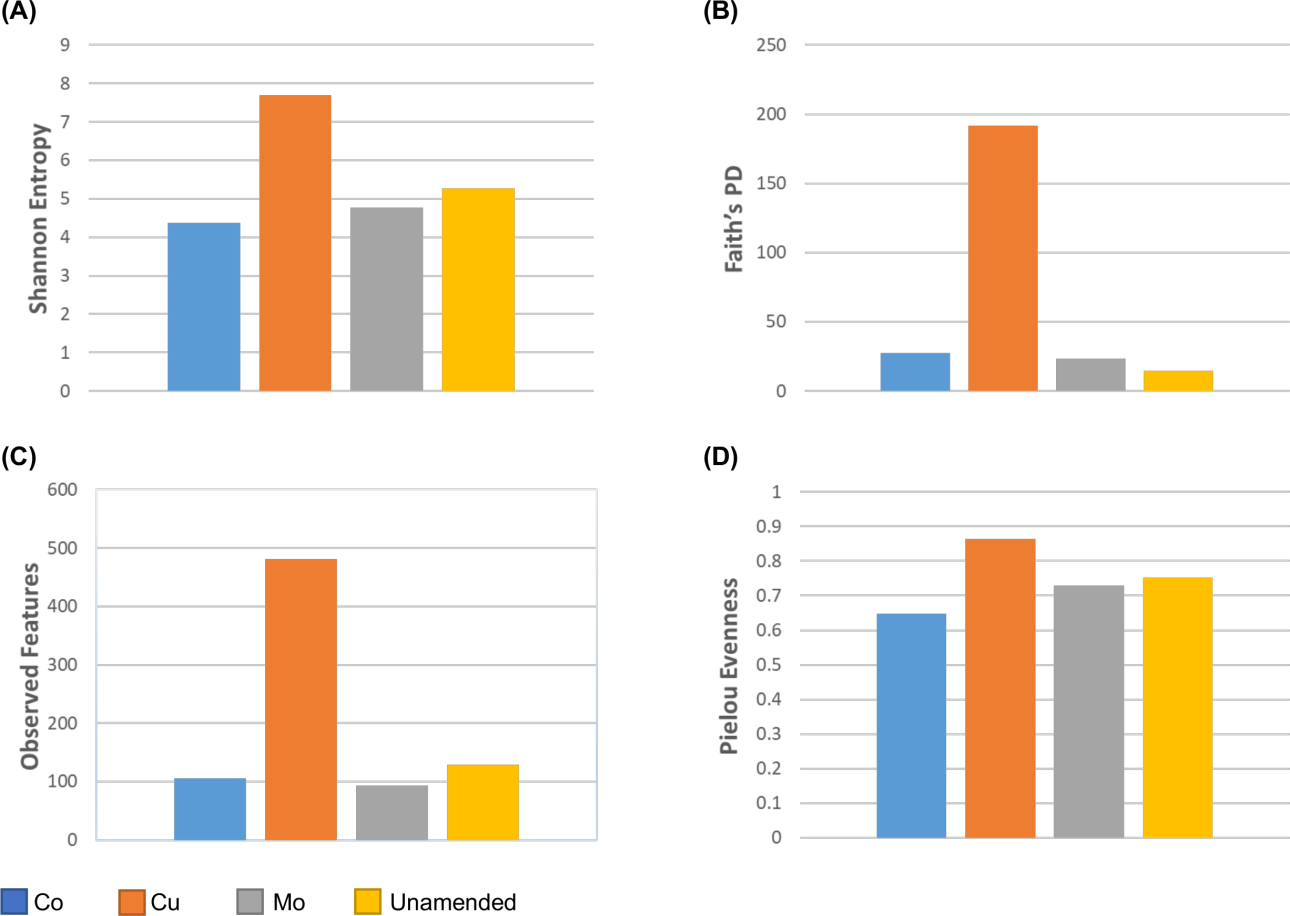
Alpha diversity of bacterial communities in unamended and in Co-, Cu-, and Mo-amended CBM microcosms.

In the Cu-amended microcosm, a significant increase of active members of *Methanosaeta*, an acetotrophic methanogen, corresponded well to a significant increase of the genus *Acetobacterium*, an acetogenic, mainly homoacetogenic group. Also, a significant increase of members of the *Lachnoclostridum* group (anaerobic, spore-forming bacteria in the order Eubacteriales that ferment diverse polysaccharides to butyrate, acetate, and ethanol) might contribute to an increase of the members of the genus *Methanosaeta* (Fig. 2 and 3). It is interesting that *Acidovorax* was not detected in Cu-amended microcosm, while it was observed in all other microcosms (Fig. 3). Possibly, Cu-amendment repressed members of the *Acidovorax* group and as a result other groups, which are usually not abundant, including *Ferribacterium* became active. The higher Faith’s PD value in Cu-amended microcosms indicates that more diverse groups are phylogenetically more distant in Cu-amended microcosm (Fig. 6). Also, significantly higher Shannon entropy in Cu-amended microcosm suggests an increase of microbial diversity after Cu-amendment. Since the substrates (H_2_, acetate, and methylated compounds, etc.) required by methanogens are products of fermentative and syntrophic bacteria, TEs added to overcome the TE limitation in this anaerobic biodegradation system may indirectly control methane production even if they do not directly affect methanogens. To date, no Cu-containing enzyme-associated with methanogenesis has been reported.

In Mo-amended microcosms, the significant increase of the hydrogenotrophic methanogens *Methanobacterium* and *Methanobrevibacter* might be due to an increase of *Actinotalea*, a petroleum hydrocarbon-degrading member of the Actinobacteria and *Lachnoclostridum,* a polysaccharide-fermenter. Some *Actinotalea* spp. may be involved in the degradation of accumulated microbial biomass in coal seams, providing fermentation products to other members of the microbial community that degrade coal to methane (11). In Co-amended microcosm, a significant increase of *Methanosarcina*, which utilizes acetate, hydrogen, and some other substrates, was observed (Fig. 2 and 3). This increase of *Methanosarcina* might be due to an increase of *Desulfuromonadaceae* spp., which can contribute to the regulation of hydrogen levels in microcosms. This bacterial group plays important roles in the degradation of organic matter and is involved in syntrophic associations especially with methanogens (114).

Both *Methanosarcina* spp. and *Methanosaeta* spp. are acetotrophic methanogens and may compete for acetate. *Methanosarcina* spp. has a metabolic advantage over *Methansaeta* spp. because *Methanosarcina* spp. can also utilize other substrates such as hydrogen, formate, and methylated compounds. In addition, *Methanosarcina* spp. have higher growth rates, but a lower affinity for acetate. The higher thresholds for acetate utilization of *Methanosarcina* spp. compared to any known *Methanosaeta* spp. provides a unique niche for members of the *Methanosaeta* (115, 116). Janssen (117) reported that *Methanosaeta* spp. can grow in a mixed enrichment culture containing acetone- and isopropanol-utilizing bacteria with a low acetate concentration which is below the threshold required for the growth of *Methanosarcina* spp. This growth advantage of *Methanosaeta* spp. over *Methanosarcina* spp. at low acetate concentrations enabled the selective enrichment of *Methanosaeta* spp. from a mixed microcosm.

Methane production from coal can be enhanced by optimally adjusting the concentrations of required TEs, but the availability of TEs *in situ* within CBM wells must be determined in further study. Our results demonstrated that increased concentrations of essential TEs caused microbial community shifts, which suggests that bioavailable TE concentrations in the subsurface coalbed environment is a driving factor for both shaping active microbial community structure and metabolic activity.

Future studies should investigate intermediate products of coal fermentation to explain if TE amendments stimulated coal fermentation and, thus, indirectly made more substrates available for methanogenesis. Furthermore, the combination of Co, Cu, and Mo in pairs or all three should be included as one of the next experiments. These studies could reveal not only how community structure might be influenced but also how methane production is further enhanced or limited.

### Conclusions

In this study, we hypothesized that selected TEs affect the structure and function of active bacterial and methanogenic communities in subsurface coalbeds, with the ultimate goal to stimulate methane production. Three of eight tested TEs significantly enhanced methane production, which correlated with higher transcript levels of *mcrA*, the gene encoding the final step in methanogenesis. TE amendment resulted in compositional shifts of the active methanogenic and the active bacterial communities. The findings of this study yield new insights to not only identify possible limitations of microbes by replenishment of TEs within their specific hydrological placement but also into the importance of essential TEs for the metabolic activity of microbial communities involved in subsurface coalbed methane production and provides a better understanding of how microbial community composition can be shaped by availability of certain TEs. These findings contribute to future research efforts for enhancement of coalbed methane production in the field.

## Data Availability

The nucleotide sequence data and metadata reported in this study were deposited in GenBank with the accession numbers PP067190-PP067334.

## SUPPLEMENTAL MATERIAL

Fig. S1 to S5 and Tables S1 and S2 spectrum.03508-23-s0001.pdf The supplemental material contains 2 tables and 5 figures, plus references used exclusively in the supplemental material.

ASM does not own the copyrights to Supplemental Material that may be linked to, or accessed through, an article. The authors have granted ASM a non-exclusive, world-wide license to publish the Supplemental Material files. Please contact the corresponding author directly for reuse.
