# Supplementary material for: Selective trace elements significantly enhanced methane production in coal bed methane systems by stimulating microbial activity
Source: Microbiol Spectr. 2024 Jan 18;12(2):e03508-23. doi: 10.1128/spectrum.03508-23 (PMC10846109; doi:10.1128/spectrum.03508-23)
Supplement: Fig. S1 to S5 and Tables S1 and S2 — The supplemental material contains 2 tables and 5 figures, plus references used exclusively in the supplemental material. [file spectrum.03508-23-s0001.pdf]

Supplemental Materials for

Selective trace elements significantly enhanced methane production in Coal Bed Methane systems by stimulating microbial activity

Kuk-Jeong Chin, Burcu Ünal, Michael Sanderson, Feranmi Aboderin, Klaus Nüsslein

This file includes:

1. Tables S1 – S2
2. Figures S1 – S5
3. References used in Supplemental Materials

1. Supplemental Tables

Table S1. *In situ* trace element concentrations in water produced from eleven CBM wells immediately after pumping it to the surface within the Tongue River member of the Fort Union Formation near Gillette, Wyoming. Samples were first collected from eight different CBM wells in 2008 (Well 1-8) to determine the range of potentially stimulating TE concentrations. Three additional CBM wells (Wells A-C) were sampled for produced water in 2012 to set up the microcosms used in this study.

Trace element concentrations in well production water (μg/L)

Well	Mo	Co	Cu	Zn	Mn	Fe	Ni	W
Well-1	0.206	0.014	0.028	11.070	10.920	604.400	0.060	BDL
Well-2*	0.617	0.031	0.479	21.610	6.846	14.920	0.756	0.037
Well-3	0.756	0.034	8.792	17.590	26.230	3.503	0.886	0.052
Well-4	0.475	0.099	0.344	6.269	8.483	921.500	2.088	0.157
Well-5	0.818	0.125	3.967	18.650	9.618	BDL	0.681	0.036
Well-6	0.117	0.019	0.484	5.991	11.280	333.100	0.365	BDL
Well-7	0.146	0.021	1.520	5.625	6.429	97.870	0.191	BDL
Well-8	0.060	0.032	0.027	7.507	12.860	BDL	1.245	BDL
Average 1-8 (SD)	0.399 (0.30)	0.047 (0.04)	1.955 (3.05)	11.789 (6.52)	11.583 (6.31)	329.216 (370.11)	0.784 (0.65)	0.071 (0.05)
Well-A	0.066	0.153	6.186	204.954	12.471	57.028	5.810	BDL
Well-B #	14.607	1.053	2.866	305.805	17.285	20.791	79.470	BDL
Well-C	0.176	0.134	4.924	114.035	2.282	49.802	28.818	BDL
Average A-C (SD)	4.949 (8.36)	0.446 (0.52)	4.659 (1.68)	208.265 (95.93)	10.679 (7.66)	42.540 (19.18)	38.032 (37.68)	BDL

*: Produced water from this well was used in a previous study for enrichment cultures (Ünal *et al*., 2012).

#: Produced water sample was used for the final set up of microcosms.

BDL, below detection limit. SD, standard deviation

Table S2. High-affinity transporters for Co, Cu, and Mo, their major biological uses, and the respective metalloproteins in Bacteria and Archaea derived from the current literature.

Trace Elements	High Affinity Transporters	Major User Forms	Metalloproteins	Reference
**Co**	Cbi/NikMNQO<br>NikABCDE<br>NiCoT<br>HupE/UreJ<br>UreH<br>Other Co transporters (CbtAB, CbtC, CbtD, CbtE, CbtF, CbtG and CbtX)	B12	• Methylmalonyl-CoA mutase<br>• Isobutyryl-CoA mutase<br>• Ethylmalonyl-CoA mutase<br>• Glutamate mutase<br>• Methyleneglutarate mutase<br>• D-lysine 5,6-aminomutase<br>• Diol dehydratase<br>• Glycerol dehydratase<br>• Ethanolamine ammonia lyase<br>• B12-dependent ribonucleotide reductase<br>• Methionine synthase<br>• Other methyltransferases: Mta, Mtm, Mtb, Mtt, Mts and Mtv<br>• B12-dependent reductive dehalogenase CprA	Daas *et al*. 1996<br>DiMarco *et al*. 1990<br>Hagemeier *et al*. 2006<br>Harris *et al.* 2008.<br>Lin *et al.* 1989<br>Paulo *et al*. 2017<br>Wintsche *et al.* 2016
**Cu**	CtaA (importer)<br>CopA (exporter)<br>CutC (exporter)<br>CusCBA/CFBA (exporter)	Cu	• Plastocyanin family (incl. plastocyanin, amicyanin, pseudoazurin, halocyanin)<br>• Azurin family (incl. azurin and auracyanin)<br>• Rusticyanin<br>• Nitrosocyanin<br>• Cytochrome c oxidase subunit I and II<br>• Nitrous oxide reductase<br>• NADH dehydrogenase 2<br>• Cu-Zn superoxide dismutase<br>• Copper amine oxidase<br>• Particulate methane monooxygenase<br>• Nitrite reductase<br>• Multicopper oxidases (CueO, laccase, bilirubin<br>• oxidase, phenoxazinone synthase, etc.)<br>• Tyrosinase	Hallam *et al.* 2003

**Mo**	ModABC<br>WtpABC<br>TupABC	Moco	• Xanthine oxidase<br>• Sulfite oxidase<br>• Dimethylsulfoxide reductase<br>• Aldehyde:ferredoxin oxidoreductase	Durfor *et al*. 1983<br>Kessler *et al.* 1997<br>Kniemeyer and Heider 2001<br>Murray and van den Berg, L., 1981.<br>Scherer, 1988<br>Vorholt, and Thauer, 2002

2. Supplemental Figures

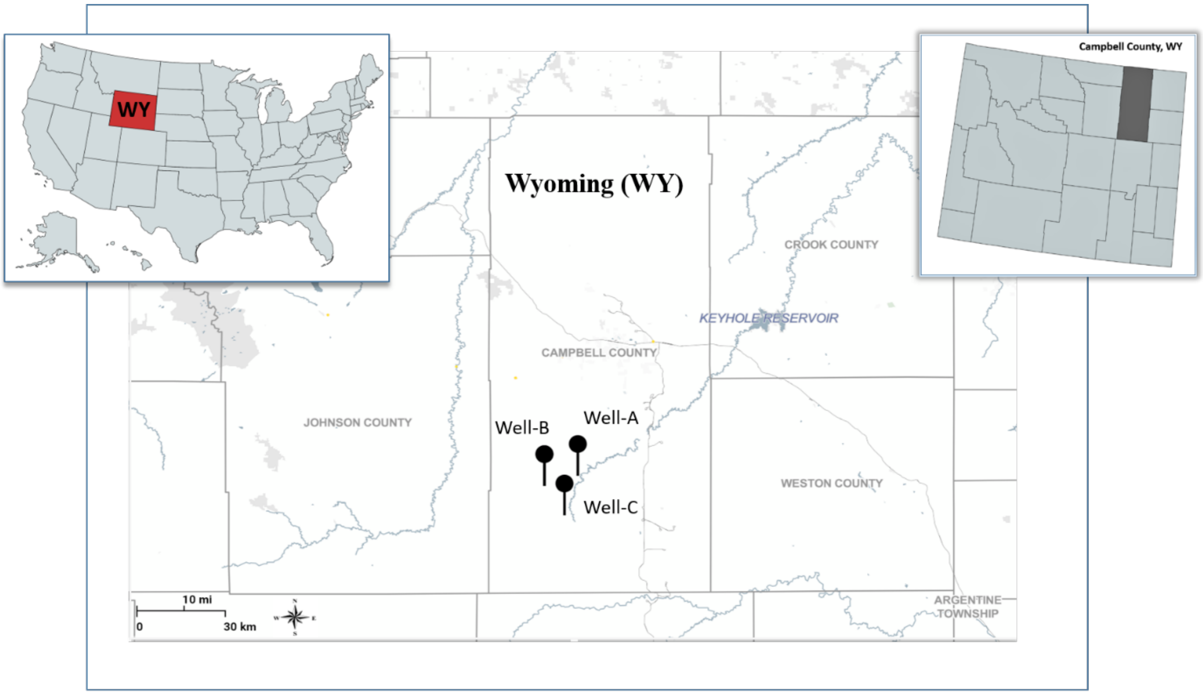

Figure S1: Locations of three CBM wells (Well-A, Well-B, Well-C) in the Powder River Basin, Wyoming, USA. Sub-bituminous coal collected from the water sampling site (Wyodak-Anderson Coal Zone) and the produced water sampled from Well-B were used for TE-amended microcosms.

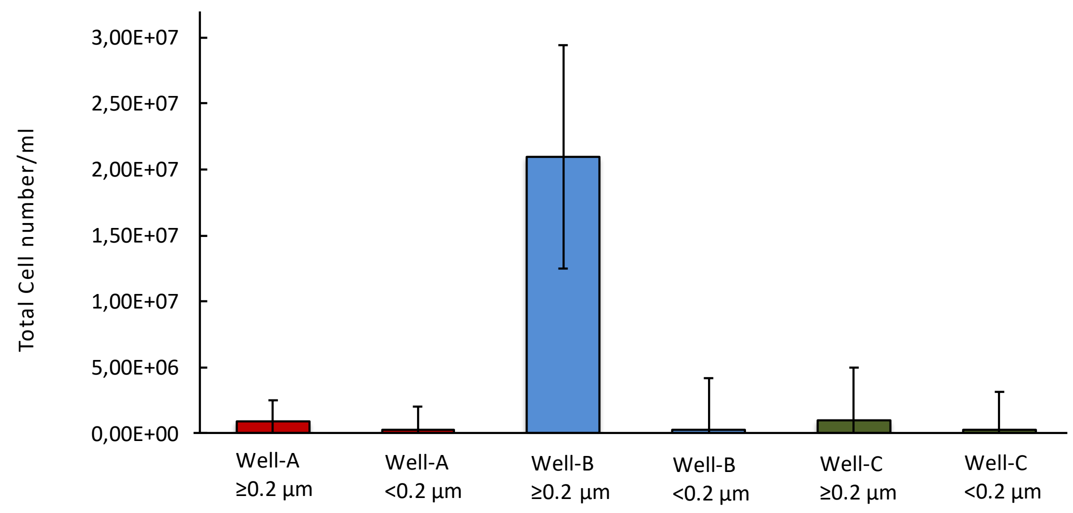

Figure S2: Average total microbial cell numbers estimated based on total direct counts by DAPI staining. Cell size fractions were separated by differential filtration to compare if all wells were similarly oligotrophic. Data are presented for a sample larger than 0.2 µm in diameter (Well-B ≥ 0.2 µm), and a second sample that passed through a filter with a pore size of 0.2 µm and was collected on a filter with 0.1 µm pore diameter (Well-B < 0.2 µm). Error bars indicate standard deviation (n=40).

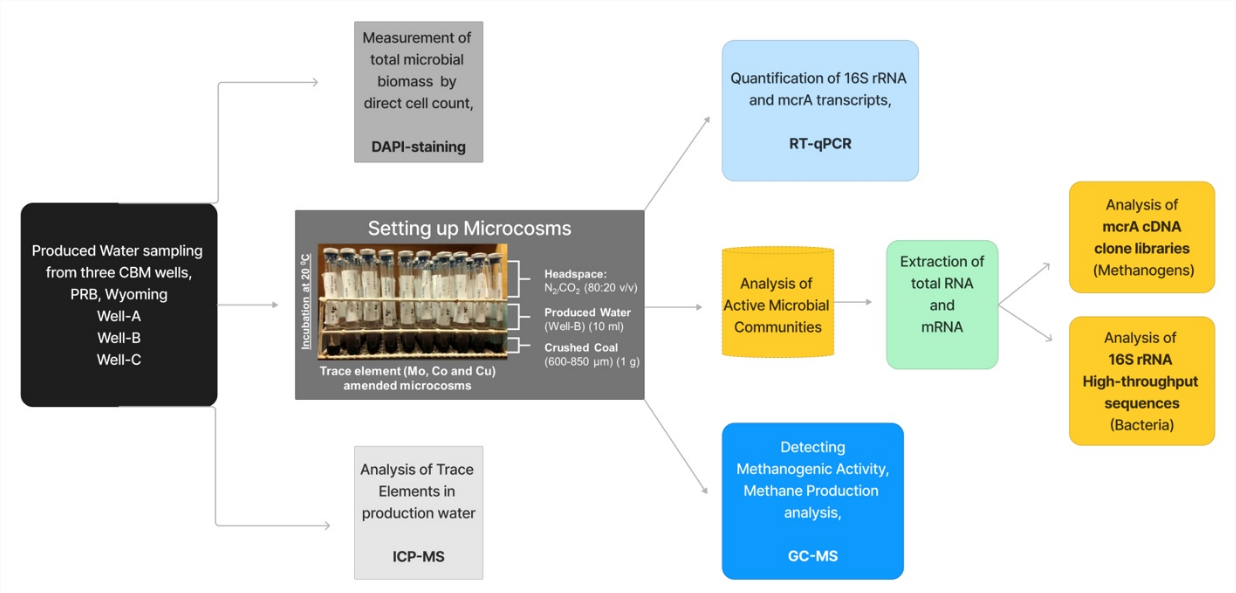

Figure S3. Schematic flow chart of the experimental design used for the establishment and monitoring of the TE-amended microcosms. The produced water samples from three CBM wells were analyzed for total cell counts (DAPI staining) and trace element concentrations (ICP-MS). Microcosms containing 10 ml CBM produced water from Well-B, supplemented with different concentrations of Co, Cu, or Mo, individually contained 1 g of crushed coal (600-850 μm in diameter) as an energy and carbon source. The headspace of the microcosm Balch tubes was replaced with a N_2/CO_2 (80:20 v/v) mixture. Microcosms treated with effective concentrations of Co, Cu, and Mo that produced the highest cumulative methane production (GC-MS) were selected for subsequent microbial analysis. The response of bacterial and methanogenic activity to trace element addition was determined by quantification of transcripts of 16S rRNA and *mcrA*, respectively (RT-qPCR). To understand how the methanogenic and bacterial communities were affected by TE addition, the *mcrA* cDNA clone library and the 16S rRNA were determined via high-throughput sequencing.

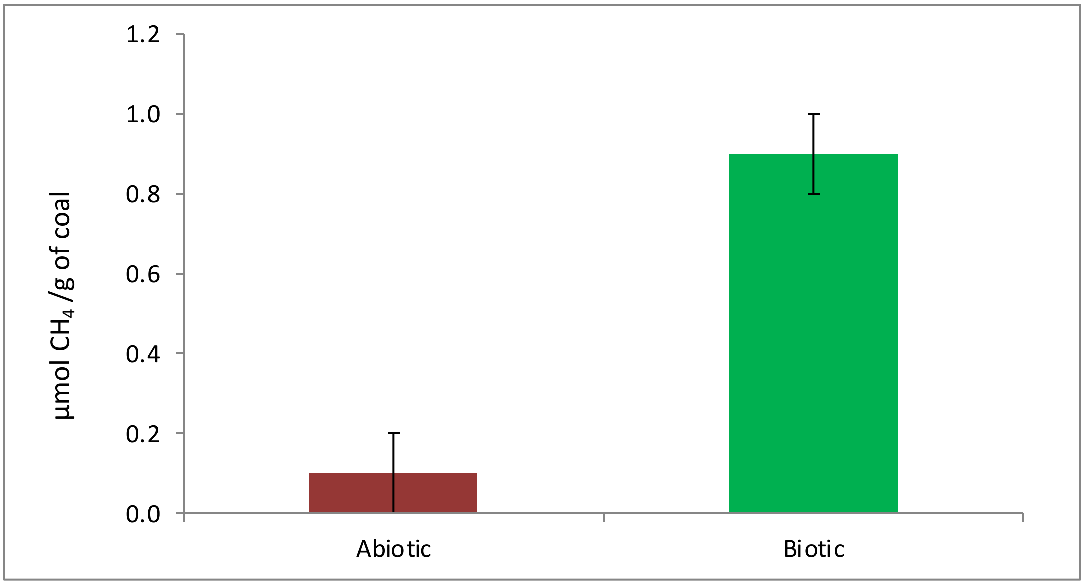

Figure S4. Methane production from coal incubated 1:1 (w:vol) in produced water (biotic control) next to a parallel incubation with an abiotic control (filter sterilized). Cumulative methane production was significantly higher (~10x) in biotic incubations compared to the abiotic control, which represents the release of fossil gas from coal and produced water. Error bars indicate standard deviations (n=3).

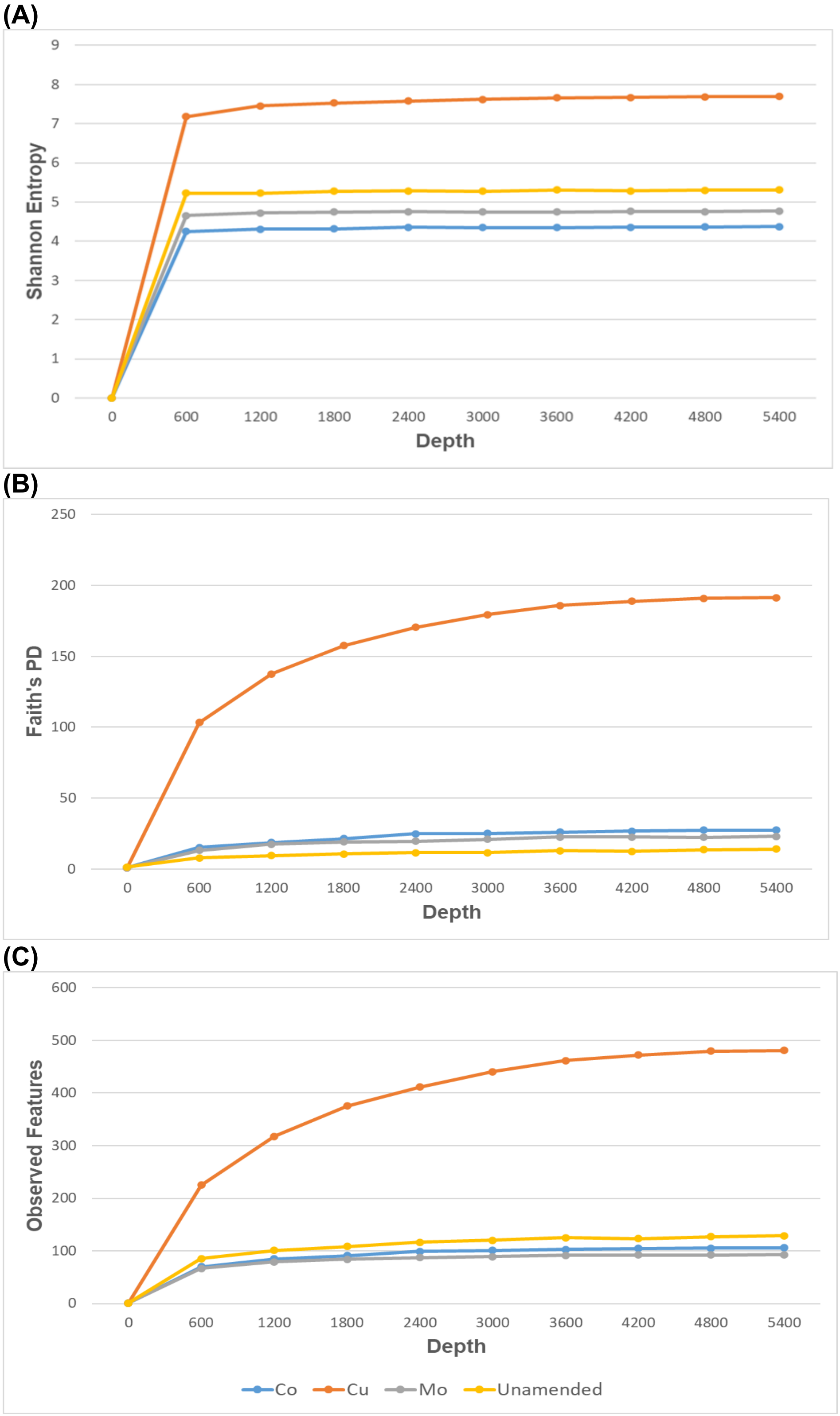

Figure S5. Rarefaction curves for alpha diversity indices of bacterial communities in unamended and in Co-, Cu- and Mo-amended CBM microcosms

3. References used in Supplemental Materials

Daas PJ, Wassenaar RW, Willemsen P, Theunissen RJ, Keltjens JT, van der Drift C, Vogels GD. 1996. Purification and properties of an enzyme involved in the ATP-dependent activation of the methanol: 2-mercaptoethanesulfonic acid methyltransferase reaction in *Methanosarcina barkeri*. *Journal of Biological Chemistry* 271(37):22339-22345.

DiMarco AA, Bobik TA, Wolfe RS. 1990. Unusual coenzymes of methanogenesis. *Annual review of biochemistry* 59(1):355-94.

Durfor CN, Wetherbee PJ, Deaton JC, Solomon EI. 1983. Characterization and spectroscopic properties of reduced Mo and W formate dehydrogenase from *C. thermoaceticum*. *Biochemical and Biophysical Research Communications* 115(1):61-67.

Hagemeier CH, Kr̈er M, Thauer RK, Warkentin E, Ermler U. 2006. Insight into the mechanism of biological methanol activation based on the crystal structure of the methanol-cobalamin methyltransferase complex. *Proceedings of the National Academy of Sciences* 103(50):18917-18922.

Hallam SJ, Girguis PR, Preston CM, Richardson PM, DeLong EF. 2003. Identification of methyl coenzyme M reductase A (mcrA) genes associated with methane-oxidizing archaea. *Applied and environmental microbiology* 69(9):5483-5491.

Harris SH, Smith RL, Barker CE. 2008. Microbial and chemical factors influencing methane production in laboratory incubations of low-rank subsurface coals. *International Journal of Coal Geology* 76(1-2):46-51.

Kessler PS, McLarnan J, Leigh JA. 1997. Nitrogenase phylogeny and the molybdenum dependence of nitrogen fixation in *Methanococcus maripaludis*. *Journal of bacteriology* 179(2):541-3.

Kniemeyer O, Heider J. 2001. Ethylbenzene dehydrogenase, a novel hydrocarbon-oxidizing molybdenum/iron-sulfur/heme enzyme. *Journal of Biological Chemistry* 276(24):21381-6.

Lin DG, Nishio N, Mazumder TK, Nagai S. 1989. Influence of Co 2+, Ni 2+ and Fe 2+ on the production of tetrapyrroles by *Methanosarcina barkeri*. *Applied microbiology and biotechnology* 30:196-200.

Murray WD, Van Den Berg L. 1981. Effects of nickel, cobalt, and molybdenum on performance of methanogenic fixed-film reactors. *Applied and environmental microbiology* 42(3):502-5.

Paulo LM, Ramiro-Garcia J, van Mourik S, Stams AJ, Sousa DZ. 2017. Effect of nickel and cobalt on methanogenic enrichment cultures and role of biogenic sulfide in metal toxicity attenuation. *Frontiers in microbiology* 8:1341.

Scherer P. 1988. Vanadium and molybdenum requirement for the fixation of molecular nitrogen by two *Methanosarcina* strains. *Archives of microbiology* 151(1):44-8.

Ünal B, Perry VR, Sheth M, Gomez-Alvarez V, Chin KJ, Nüsslein K. 2012. Trace elements affect methanogenic activity and diversity in enrichments from subsurface coal bed produced water. *Frontiers in microbiology* 3:175.

Vorholt JA, Thauer RK. 2002. Molybdenum and tungsten enzymes in C1 metabolism. *Metal ions in biological systems.* eds A. Sigel and H. Sigel (Basel, NY: Marcel Dekker), 39:571–603.

Wintsche B, Glaser K, Sträuber H, Centler F, Liebetrau J, Harms H, Kleinsteuber S. 2016. Trace elements induce predominance among methanogenic activity in anaerobic digestion. *Frontiers in microbiology* 7:2034.
